# Identification of a functionally significant tri-allelic genotype in the Tyrosinase gene (*TYR*) causing hypomorphic oculocutaneous albinism (OCA1B)

**DOI:** 10.1038/s41598-017-04401-5

**Published:** 2017-06-30

**Authors:** Chelsea S. Norman, Luke O’Gorman, Jane Gibson, Reuben J. Pengelly, Diana Baralle, J. Arjuna Ratnayaka, Helen Griffiths, Matthew Rose-Zerilli, Megan Ranger, David Bunyan, Helena Lee, Rhiannon Page, Tutte Newall, Fatima Shawkat, Christopher Mattocks, Daniel Ward, Sarah Ennis, Jay E. Self

**Affiliations:** 1 Clinical and Experimental Sciences, Faculty of Medicine, University of Southampton, Southampton, UK; 2 Human Development and Health, Faculty of Medicine, University of Southampton, Southampton, UK; 3 Biological Sciences, Faculty of Natural and Environmental Sciences, University of Southampton, Southampton, UK; 4 Human Genetics & Genomic Medicine, Faculty of Medicine, University of Southampton, Southampton, UK; 5 Cancer Sciences Unit, Faculty of Medicine, University of Southampton, Southampton, UK; 6 Eye Unit, University Hospital Southampton, Southampton, UK; 7 Molecular Genetics Wessex Regional Genetics Laboratory, Salisbury NHS Foundation Trust, Salisbury, UK; 8 Wessex Investigational Science Hub, University Hospital Southampton, Southampton, UK

## Abstract

Oculocutaneous albinism (OCA) and ocular albinism (OA) are inherited disorders of melanin biosynthesis, resulting in loss of pigment and severe visual deficits. OCA encompasses a range of subtypes with overlapping, often hypomorphic phenotypes. OCA1 is the most common cause of albinism in European populations and is inherited through autosomal recessive mutations in the Tyrosinase (*TYR)* gene. However, there is a high level of reported missing heritability, where only a single heterozygous mutation is found in *TYR*. This is also the case for other OCA subtypes including OCA2 caused by mutations in the *OCA2* gene. Here we have interrogated the genetic cause of albinism in a well phenotyped, hypomorphic albinism population by sequencing a broad gene panel and performing segregation studies on phenotyped family members. Of eighteen probands we can confidently diagnose three with OA and OCA2, and one with a *PAX6* mutation. Of six probands with only a single heterozygous mutation in *TYR*, all were found to have the two common variants S192Y and R402Q. Our results suggest that a combination of R402Q and S192Y with a deleterious mutation in a ‘tri-allelic genotype’ can account for missing heritability in some hypomorphic OCA1 albinism phenotypes.

## Introduction

Oculocutaneous albinism (OCA) and X-linked ocular albinism (OA) are inherited disorders of melanin biosynthesis which result in varied levels of hypopigmentation of skin, hair, and ocular tissues^1^. Characteristic ophthalmic features include reduced visual acuity, nystagmus, strabismus, and photophobia. Closer examination may reveal foveal hypoplasia (abnormal retinal development), asymmetry of visual evoked potential (VEP) responses, and iris transillumination^1^. Foveal hypoplasia for instance, can be determined using Spectral-Domain Optical Coherence Tomography (SD-OCT) and then graded on a scale of 1–4 (Thomas *et al*.^2^), and the asymmetry of visual-evoked potentials documents the excessive decussation at the optic chiasm seen in albinism^3^. Partial phenotypes are described widely in the literature in which some features are present but others are lacking (e.g. nystagmus or foveal hypoplasia), however, phenotyping methods have varied significantly and the partial phenotype has never before been described in detail^4–6^. Current management of albinism focusses on correction of any refractive errors, management of head postures/strabismus and on the importance of effective sun protection. Another important factor in the management albinism is genetic counselling; therefore it is important to confirm a genetic diagnosis for patients.

Six genes involved in melanin biosynthesis pathway are known to cause forms of OCA and OA: *TYR* (tyrosinase), *OCA2, TYRP1* (tyrosinase-like protein 1), *SLC45A2* (solute carrier family 45 member 2), *SLC24A5* (solute carrier family 24 member 5), and *C10orf11* (chromosome 10 open reading frame 11) accounting for OCA subtypes 1–4 and 6–7 respectively, and *GPR143* accounting for OA1^6^, see Table 1. All of the OCA subtypes are understood to be inherited as autosomal recessive disorders but the subtypes are heterogeneous in pigmentary phenotype^1, 7, 8^. OCA1 has a mixed phenotype and is further split into OCA1A and OCA1B. OCA1A describes complete loss of tyrosinase activity (previously described as ‘tyrosine negative’ albinism) and is characterised by an apparent total lack of pigment. Some tyrosinase function is retained in OCA1B, allowing pigment to accumulate and generate a phenotype of minimal to near normal skin pigmentation, as is also the case for the other described OCA and OA phenotypes^1, 8^. Phenotypes of partial OCA also overlap with those seen in patients with dominant mutations in the *PAX6* gene, which is involved in ocular development, where a variety of phenotypes have been described including foveal hypoplasia, iris trans-illumination and nystagmus^9^.

**Table 1:** Table to describe HGNC approved gene names associated with the subtypes of OCA and OA.

HGNC symbol	HGNC name	Albinism subtype	Mode of inheritance
*TYR*	Tyrosinase	OCA1A	Autosomal recessive
		OCA1B	
*OCA2* (*P* gene)	OCA2 melanosomal transmembrane protein	OCA2	Autosomal recessive
*TYRP1*	Tyrosinase related protein 1	OCA3	Autosomal recessive
*SLC45A2*	Solute carrier family 45 member 2	OCA4	Autosomal recessive
*—*	Chromosomal location 4q24	OCA5	Autosomal recessive
*SLC24A5*	Solute carrier family 24 member 5	OCA6	Autosomal recessive
*C10orf11*	Chromosome 10 open reading frame 11	OCA7	Autosomal recessive
*GPR143*	G protein-coupled receptor 143	OA1	X-linked recessive

OCA5 has been attributed to a chromosomal location but does not yet have an associated gene^46^.

As the most severe form of OCA, OCA1A is often recognised in early infancy. King *et al*. proposed that white hair from birth can be used to predict OCA1^8^, with 85% of patients identified in this way testing positive for pathogenic *TYR* mutations. However, 15% of OCA cases identified in this way had no accountable genetic mutation, and 29% of those confirmed as OCA1 had only one identifiable *TYR* mutation^8^. It is widely recognised that the OCA genes do not account for all non-syndromic cases, as many as 30% of OCA1A occurrences have an unknown genetic origin^10, 11^ and this percentage may be higher for cases of partial albinism^12^. It is also important to note that a variety of techniques have been employed to screen for tyrosinase gene mutations in these studies and no method has 100% sensitivity.

An individual’s pigmentary phenotype depends on polymorphisms in many genes, including polymorphisms in the OCA genes^13–15^. Ethnic background may play a large role in an individual’s susceptibility to the albinism phenotype, with hypomorphic mutations having a more damaging effect on a less active pigmentary pathway^16, 17^. It has been suggested that inheritance of OCA2 is not purely recessive, with the example of haploinsufficiency noticeably affecting skin complexion in a Hispanic family, arguably due to the already fair skin tone^13^. It has also been suggested that a synergistic interaction between genes throughout the pigment pathway may exist in albinism phenotypes, evidenced by one family exhibiting an OCA2 phenotype that is modified by a mutation in the gene for OCA3^14^ and a correlation between *OCA2* and *MC1R* variants in a small albinism cohort^18^. The quantitative effect of pigmentation also has relevance to OCA1B, particularly the notion of autosomal recessive ocular albinism (AROA), an arbitrary characterisation that has been used previously to describe cases with clinically mild OCA1B^19, 20^.

AROA sparked a debate over the possible pathogenicity of two *TYR* polymorphisms, rs1126809 (p.R402Q) and rs1042602 (p.S192Y), common in Caucasian populations with allele frequencies ~28–36%^21^. Functional studies have shown the R402Q polymorphism produces a thermolabile enzyme, retained by the cells endoplasmic reticulum, with a 75% reduction in catalytic activity compared to the wild-type^15, 22, 23^; and S192Y results in a 40% reduction of tyrosinase enzymatic activity^24^. Multiple OCA1 studies have shown the R402Q allele is strongly associated with albinism patients with only one mutation^12, 17, 20^.

R402Q has been proposed as a causal variant, though only when inherited on the trans allele to a null activity *TYR* mutation^19, 20^. However this was disputed with evidence of no OCA phenotype in the parents of affected probands even when they carried a combination of null mutation and R402Q^25^. This has led to the question of whether it is possible for an additional variant to be necessary for manifestation of the ocular phenotype. The combination of two common variants may produce a reduction in TYR activity that, when co-inherited with a deleterious *TYR* mutation, provides sufficient loss of activity to cause an albino phenotype^15, 16^. A similar tri-allelic hypothesis has been demonstrated in Bardet-Biedl syndrome^26^, but is yet to be demonstrated in albinism.

In this study, we have sequenced all the known albinism genes in patients with possible hypomorphic albinism phenotypes, identified through detailed ocular phenotyping in a tertiary eye clinic. Probands with some, but not all of the typical cutaneous and ocular features of OCA1A were defined as having a likely hypomorphic albinism phenotype. For the first time, we investigate common variants in tri-allelic pattern of inheritance using detailed phenotyping and segregation studies in relatives to identify the causative genotype.

## Methods

Patients were recruited following the tenets of the declaration of Helsinki, informed consent was obtained and the research was approved by the Southampton & South West Hampshire Research Ethics Committee.

We investigated the genetic cause of eighteen probands categorized as having hypomorphic albinism. Probands were identified from a regional paediatric nystagmus clinic. All patients seen in this clinic underwent detailed phenotyping of skin and hair tone in context of family pigmentation, orthoptic examination, anterior and posterior segment examinations on a slit-lamp biomicroscope, electrodiagnostics including an electroretinogram (ERG) and visual evoked potential (VEP), and optical coherence tomography (OCT) of the macular using either a Leica OCT system or a Spectralis OCT (Heidelberg Engineering). Eye movement recordings were made on an EYElink10000 + (SR research) eye tracker and refraction was measured. Saliva was collected and DNA extracted using Oragene-DNA kit (OG-575)(DNA Genotek).

Probands with at least two phenotypic features of albinism (skin and hair pigmentation deemed to be low within the family context/nystagmus/foveal hypoplasia/VEP crossing/iris transillumination) as determined by a consultant ophthalmologist (JES), were chosen from a larger database containing approximately 300 probands with albino and/or nystagmus phenotypes. Probands were additionally excluded if they had complete characteristics of OCA1A or where DNA quality was poor.

The DNA samples were enriched using the TruSight One capture platform (Illumina 5200 Illumina Way San Diego, California USA). TruSight One has been dubbed a “clinical exome”, covering 4813 genes associated with disease-causing mutations. The panel targets and captures most of the coding regions of OCA genes 1–4 & 6, the OA1 gene, all syndromic albinism genes and *PAX6*, coverage of genes is shown in Supplementary Table 1. Prepared libraries underwent paired-end sequencing on an Illumina NextSeq 500 machine.

Next generation sequencing (NGS) data was aligned against the human reference genome (hg19) using Novoalign (v2.08.02). The mean read depth across all samples was 167 (Supplementary Table 1) with 97.2% of all target regions achieving a depth of 20X or greater. Variant calling was performed using SAMtools v0.1.19^27^ and variant annotation using ANNOVAR^28^ against RefSeq transcripts. Additional annotation was applied using the Human Gene Mutation Database, HGMD^29^. The mean depth and percentage of target captured at a read depth of 20X for each sample is listed in Supplementary Table 2.

Variants within the genes of interest were filtered using 1000 Genomes Project Minor Allele Frequency (MAF) (<0.05) and the *in silico* pathogenicity prediction tools SIFT (<0.05), PolyPhen2 HumVar (possibly damaging and probably damaging) and GERP++ (>2). SIFT predicts pathogenicity of missense mutations based on homology^30^, PolyPhen2 HumVar predicts pathogenicity based on conservation and protein structure/function^31^ and GERP++ measures evolutionary constraint^32^. The six probands with only a single heterozygous *TYR* mutation were further investigated. Sanger sequencing was used to confirm and segregate each *TYR* variant in probands and family members, primers used are listed in Supplementary Table 3. Primers designed by Chaki *et al*. were used to for amplification of *TYR* exon 4 to avoid amplification of the highly homologous *TYRL* gene^33^.

Multiple ligation-dependent probe amplification (MLPA) was carried out for the *TYR* and *OCA2* genes as according to the manufacturer’s instructions with the current SALSA MLPA P325 *OCA2* probe mix at the time of testing (MRC-Holland, the Netherlands). Partial albinism probands and control individuals were compared. Subsequent data were analysed using the MLPA analysis function of the GeneMarker (version 1.85) software (SoftGenetics, USA)^34^.

## Results

### Diagnosis of hypomorphic albinism

**Figure 1:**
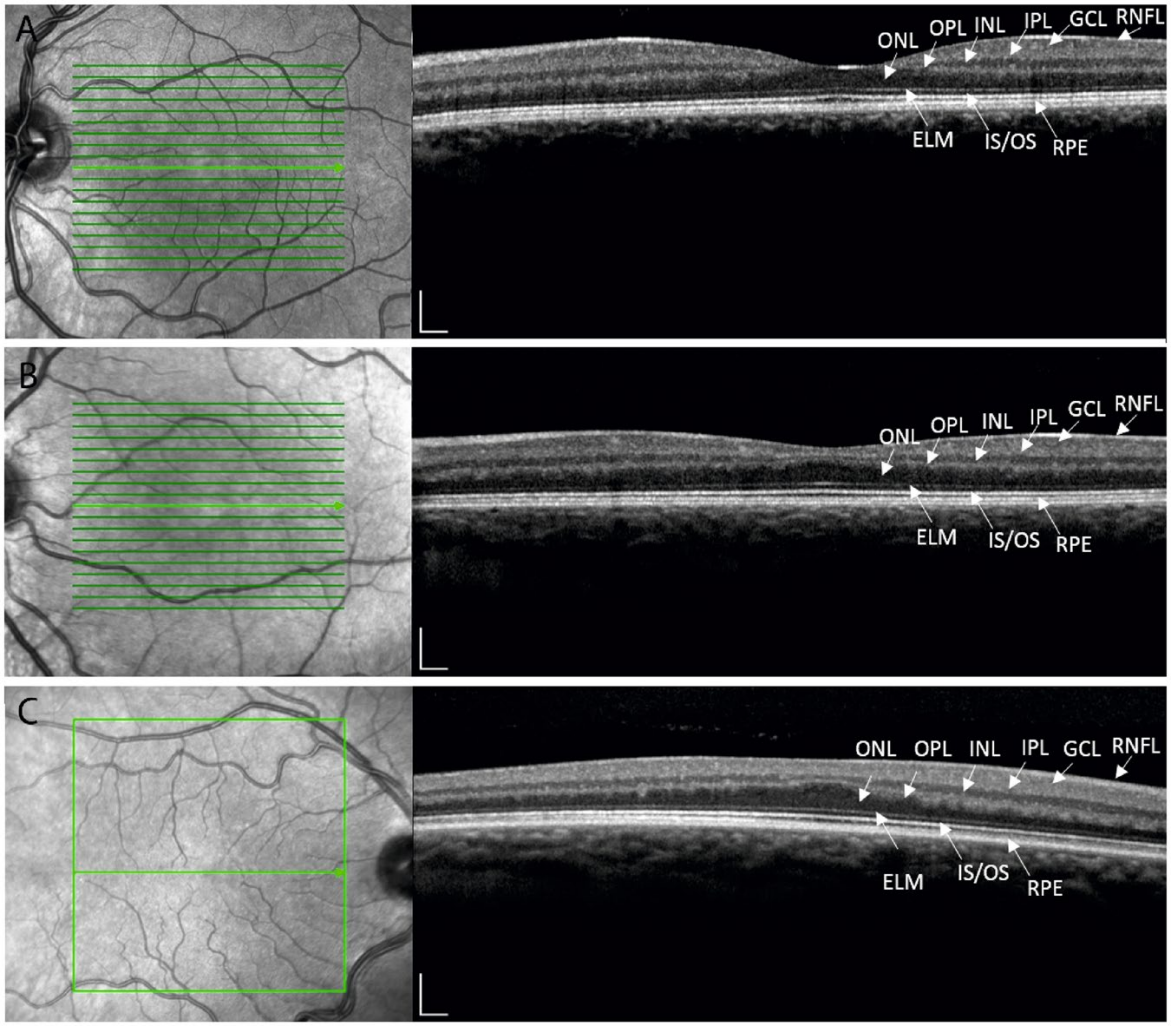
OCT images using the Heidelberg Spectralis Diagnostic imaging platform. (**a**) Normal fovea (Mother of proband 13) (**b**) Foveal hypoplasia grade 1 (brother of proband 13) (**c**) Foveal hypoplasia grade 3 (Mother of proband 18). Foveal grading according to the Thomas *et al*. grading system^2^. Outer nuclear layers (ONL), outer plexiform layers (OPL), inner nuclear layers (INL), inner plexiform layers (IPL), ganglion cell layers (GCL) and retinal nerve fibre layers (RNFL) are labelled.

The hypomorphic albinism phenotype varied in both ocular phenotype and pigment level between probands and between family members. For example proband and mother in family 3 both have a phenotype consistent with partial albinism, however the proband exhibits a severe loss of cutaneous pigment but no iris transillumination, whereas the cutaneous pigment in the proband’s mother is within that of the family context but ocular investigations revealed trans-illumination defects. The level of foveal hypoplasia also varied between patients and within families. Example OCT images taken from the cohort are in Fig. 1, demonstrating the broad range of foveal developmental anomalies identified.

NGS data for OCA genes 1–4 & 6, the OA1 gene, and *PAX6* were initially filtered using predictive scores from SIFT and PolyPhen. GERP +  + was also noted, and variants with a MAF >5% were considered benign and were filtered using the 1000 Genomes Project dataset. This revealed eighteen potentially causal mutations across five genes in thirteen probands, leaving five probands with no variants passing the filtering threshold. No proband was found to have more than three variants using this methodology, results in Table 2.

**Table 2:** Predicted causal variants, in eighteen probands with phenotypes matching hypomorphic albinism.

Proband	Variant 1	Variant 2	Variant 3
1	—	—	—
2	—	—	—
3	*TYR* c.529 G > T p.V177F (SIFT = . PolyPhen = D GERP = 5.16)	*OCA2* c.822 G > C p.W274C (SIFT = 0 PolyPhen = D GERP = 4.66)	*OCA2* c.1948C > G p.Loo650V (SIFT = 0.03 PolyPhen = D GERP = 5.75)
4	*TYR* c.1467dup p.T489fs	—	—
5	*TYR* c.505_507del p.D169del	—	—
6	*TYR* c.732_733del p.C244Ter	—	—
7	*TYR* c.1204 C > T p.R402Ter	—	—
8	*OCA2* c.1393 A > G p.N465D (SIFT = 0.01 PolyPhen = D GERP = 5.33)	*TYRP1* c.1037 C > G p.P346R (SIFT = 0 PolyPhen = D GERP = 5.73)	—
9	*TYR* c.1217 C > T p.P406L (SIFT = . PolyPhen = D GERP = 4.68)	*PAX6* c.1264 C > A p.Q422K (SIFT = 0 PolyPhen = D GERP = 6.16)	—
10	*GPR143* c.485del p.W162fs	—	—
11	—	—	—
12	*TYR* c.1217 C > T p.P406L (SIFT = . PolyPhen = D GERP = 4.68)	—	—
13	*OCA2* c.1606C > T p.R536C (SIFT = 0.01 PolyPhen = D GERP = 5.8)	—	—
14	—	—	—
15	—	—	—
16	*OCA2* c.1255 G > A p.V419I (SIFT = 0.02 PolyPhen = D GERP = 5.2)	*OCA2* c.1025 A > G p.Y342C (SIFT = 0 PolyPhen = D GERP = 5.55)	—
17	*OCA2* c.1255 G > A p.V419I (SIFT = 0.02 PolyPhen = D GERP = 5.2)	—	—
18	*TYR* c.1264 C > T p.R422W (SIFT = . PolyPhen = D GERP = 2.69)	—	—

Pathogenicity determined by filtering all variants in the genes; *TYR*, *OCA2*, *TYRP1*, *SLC45A2*, *SLC24A5*, *C10orf11* and *PAX6*, with the parameters MAF < 0.05, SIFT < 0.05, PolyPhen2 = possibly damaging or probably damaging. The prediction scores for non-synonymous variants are included, for some mutations a prediction score was not available at the time of analysis. Gene accessions number: *TYR* NM_000372, *OCA2* NM_001300984, *PAX6* NM_001258465, T*YRP1* NM_000550, *GPR143* NM_000273.

Proband 9 was found to have a likely pathogenic mutation in the *PAX6* gene and proband 10 has a deletion resulting in a frameshift mutation in the X-linked gene, *GPR143*. Probands 3 and 16 each have two compound heterozygous mutations in the *OCA2* gene, these putative variants would explain autosomal recessive inheritance of OCA2. Proband 8 has a single mutation in *OCA2* and a second mutation in *TYRP1* which would require further investigation before concluding causality. Two probands, 13 and 17, each have a single heterozygous mutation in the *OCA2* gene with no second mutation identified. Furthermore, six probands each had a single heterozygous mutation in the *TYR* gene with no further variants passing the filtering threshold. Probands 3 and 9 also have *TYR* mutations, but due to potentially causal variants in other genes, these single recessive mutations may afford probands 3 and 9 carrier status only. The *TYR* mutation P406L occurs in two probands, as does the *OCA2* mutation V419I.

MLPA of *TYR* and *OCA2* was carried out in probands 1–6 and 8–12 to search for large deletions that would be missed in the NGS data. MLPA results revealed no abnormal copy numbers, ruling out whole gene/exon deletions.

### Segregation of the OCA1 tri-allelic genotype

We further investigated the single *TYR* variants in both probands and family members (families 4–7, 12 and 18 in Table 3) using Sanger sequencing to confirm and determine segregation of variants. In total, twenty probands and family members were phenotyped and genotyped, results in Table 3. The phenotyping results of these six families suggests a total nine cases of partial albinism (six probands and three affected family members). Sanger sequencing confirmed the predicted causal variants in probands and revealed variants segregated with affected family members in every case, with three unaffected family members as carriers.

**Table 3:** Phenotype-genotype table of families with Sanger-confirmed TYR variants.

ID	Relation to proband	Abnormal pigment	Nystagmus	OCT	Trans-illumination	VEP	Genotype		
							*TYR* Variant 1	R402Q	S192Y
Family 4	**Proband**	**Yes - OCA1A**	**No**	**FH**	**No**	**Crossed**	**c.1467dup p.T489fs** ^8, 10, 20, 37^	**Het**	**Het**
	Father	No	No	Normal	No	—	c.1467dup p.T489fs^8, 10, 20, 37^	WT	WT
	Mother	No	No	Normal	No	—	WT	Het	Hom
	Sister	No	No	Normal	No	—	WT	WT	Het
Family 5	**Proband**	**Yes**	**No**	**FH**	**Yes**	**Abnormal**	**c.505_507del p.D169del** ^40^	**Het**	**Het**
	Mother	No	No	—	—	—	WT	Het	Het
	Father	No	No	—	—	—	c.505_507del p.D169del^40^	WT	Het
Family 6	**Proband**	**Yes - OCA1A**	**No**	**FH**	**No**	**Normal**	**c.732_733del p.C244Ter** ^41^	**Het**	**Het**
	**Mother**	**No**	**No**	**FH**	**Yes**	—	**c.732_733del p.C244Ter** ^41^	**Het**	**Het**
	Father	No	No	Normal	No	—	WT	Het	Het
	Sister	No	No	—	—	—	WT	Het	Het
Family 7	**Proband**	**Yes**	**Yes**	—	**Yes**	—	**c.1204 C > T p.R402Ter** ^20, 37, 38^	**Het**	**Het**
	**Sister**	**Yes**	**Yes**	—	**Yes**	—	**c.1204 C > T p.R402Ter** ^20, 37, 38^	**Het**	**Het**
	Mother	No	No	—	—	—	c.1204 C > T p.R402Ter^20, 37, 38^	WT	Het
	Father	No	No	—	—	—	WT	Het	Het
Family 12	**Proband**	**No**	**Yes**	**FH**	**No**	**Crossed**	**c.1217 C > T p.P406L** ^8, 20, 37^	**Het**	**Het**
	Mother	No	No	—	—	—	c.1217 C > T p.P406L^8, 20, 37^	WT	Het
	Grandmother	No	No	—	—	—	WT	Het	WT
Family 18	**Proband**	**Yes**	**Yes**	**FH**	**Mild**	**Inconclusive**	**c.1264 C > T p.R422W** ^8, 16, 39^	**Het**	**Het**
	**Mother**	**No**	**Yes**	**FH**	**No**	—	**WT**	**Het**	**Het**

Family number corresponds with proband number. Phenotype information (from left to right): cutaneous and hair pigmentation in context of family background, presence of nystagmus, foveal hypoplasia (FH), iris trans-illumination, and VEP asymmetry indicating (over)crossing of the optic nerve. Those with partial albinism are in bold.

**Figure 2:**
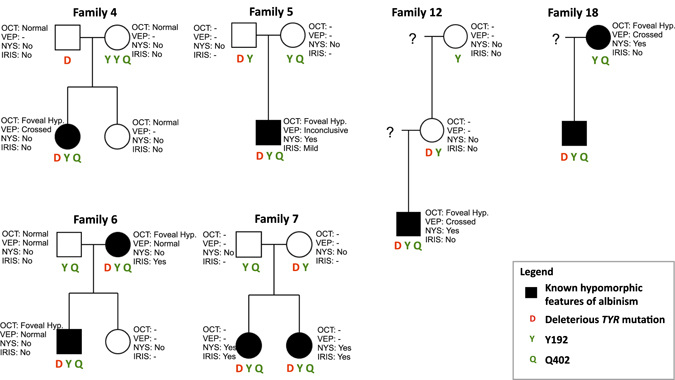
Pedigree diagrams for six families with a single *TYR* pathogenic mutation and common polymorphism phenotyping. *TYR* variants are listed beneath each family. Sanger sequencing was performed on family members as opposed to the full exonic region sequenced in probands. Family number corresponds with proband number.

To explore the apparent missing heritability in these cases we investigated the potential pathogenicity of common variants R402Q and S192Y. The NGS data was examined in probands with *TYR* mutations. All six probands were found to have both common variants. These variants were confirmed in probands with Sanger sequencing and variant segregation was determined across available members of the six pedigrees, shown in Fig. 2. The combined presence of both common polymorphisms and a putative *TYR* mutation in a tri-allelic genotype segregates with affected family members.

It can be deduced that the R402Q variant is on the *trans* allele to the deleterious *TYR* mutation in probands 4, 5, 7 and 12. In family 4 we can also be certain the S192Y variant is on the *trans* allele. The mother of proband 18 has both nystagmus and foveal hypoplasia, yet does not have the same deleterious *TYR* mutation as her son.

## Discussion

We have combined high resolution phenotyping, a broad NGS technique, segregation analysis and MLPA studies in a cohort of presumed partial albinism patients. This allows us the opportunity to perform a detailed genotype-phenotype correlation in this group of patients for the first time. In this study we identified one novel variant in the PAX6 gene, a novel frameshift variant in the GPR143 gene, two novel variants in the OCA2 gene (both in probands 3), five previously reported variants in OCA2, three novel and four previously reported variants in the TYR gene, and one previously reported variant in TYRP1 in eighteen probands. When combined, these variants provide a convincing genetic diagnosis for only 22% of our original hypomorphic albinism cohort if those with missing variants, in a presumed recessive condition (OCA1), are excluded.

The novel variant in GPR143, c.485delG, causes a frameshift mutation likely resulting in ocular albinism in proband 10. Of the six different mutations found in *OCA2*; N465D^8^, V419I, Y342C^35^ and L650V^36^ have been reported previously in association with albinism. The variants R536C and W274C are both predicted to be deleterious by SIFT, PolyPhen2 and GERP++, described in Table 2.

The probands revealed seven different mutations in the TYR gene: V177F, c.1467dup, c.505_507del, C244Ter, R422W, R402Ter and P406L. The mutation V177F has been previously reported in an albinism cohort^37^. *TYR* c.1467dup results in a frameshift and has been reported as a causal mutation multiple times^8, 10, 20, 37^. R402Ter has been reported previously and creates a premature stop codon, considered highly deleterious^20, 37, 38^. The mutation P406L has also been reported many times before in association with albinism^8, 37^, and it has been shown to reduce enzyme activity to 35%^39^. R422W has been reported as disease causing^8^, however functional studies of this mutation have conflicting results. Mondal *et al*. assayed the tyrosine hydroxylase and DOPA oxidase activity of the R422W mutant and found that the enzyme retained no activity^16^, whereas, Dolinska *et al*. assessed only DOPA oxidase activity and found that the R422W mutant retained 95% of wild-type enzyme activity. Dolinska *et al*. also state that R422W is temperature sensitive and the immature glycoprotein is degraded more quickly at 37 °C^39^, potentially accounting for the difference between assays. Reported literature ascribes many variants as disease causing throughout the coding regions of both *OCA2* and *TYR*, however recent functional studies have questioned the deleterious effect of some of these variants, particularly in the *TYR* gene^16, 39^. There is currently no functional evidence of the deleterious effect of the mutations TYR c.505_507del and TYR C244Ter though the deletions have been previously been reported as causal mutations, and the introduction of a premature stop codon is considered highly deleterious^40, 41^. It is likely that further functional analyses are necessary to produce a curated list of mutations for accurate genetic diagnosis^42^.

Six probands within our cohort were found to have single *TYR* variant previously identified in albinism patients, but no variant in another known gene. As there is no functional evidence for the variants in family 5 and family 6 there remains the possibility of another causal gene mutation. It has been suggested that this high level of missing heritability could be due to mutations in the TYR promoter or an interacting distal gene enhancer^43^. Notably, all six had also inherited R402Q and S192Y common *TYR* variants producing a tri-allelic genotype.

The common variant R402Q is located in exon 4, near to the CuB catalytic site, and produces a thermolabile enzyme^16, 22^, but it has been argued that the reduction of tyrosinase activity is not enough to produce a phenotype. The controversy over the R402Q variant stems from a paper by Oetting *et al*. which argues that segregation of R402Q with a known pathogenic variant on the homologous allele does not confer albinism^25^.

The variant S192Y is located in the CuA catalytic site of tyrosinase and has been shown to lower enzymatic activity independently to R402Q^15^. Previous studies have had stringent criteria for an OCA1 phenotype (white hair and skin and translucent irides from birth)^25^, whereas, here we have considered hypomorphic presentations that do not appear as severe but result in ocular deficits nonetheless. Here we suggest that a combination of a pathogenic mutation inherited with both variants in a tri-allelic genotype may cause a large enough reduction in tyrosinase activity for a partial OCA1 phenotype.

AROA is not an appropriate diagnosis for probands in this cohort as cutaneous and hair pigment is noticeably decreased in most probands and many family members and there is a lot of variation in ocular phenotype. Background level of pigmentation may determine the severity of the mutations as lower pigment levels will be affected more severely by the same dosage loss of tyrosinase. Therefore, our results support the theory of a causal tri-allelic genotype may go some way to account for many cases of OCA1 with apparent missing heritability. Functional studies would assist in confirming pathogenicity, thus allowing the tri-allelic genotype to be considered for both future and retrospective genetic diagnosis of OCA1.

There is potential for a double-variant haplotype, p.[S192Y;R402Q], existing on the *trans* allele to the known *TYR* mutation in affected individuals. A combination of the common variants R402Q and S192Y in *cis* may have a compound effect, producing a great enough loss of function equal to a deleterious *TYR* mutation. Each of the common variants R402Q and S192Y have a MAF of greater than 20%, and as individual SNPs they are considered benign (shown in our cohort in unaffected family members). In contrast, the predicted frequency of p.[S192Y;R402Q] in *cis* is 1.1%, using ‘British in England and Scotland’ participants of the 1000 Genomes project (GBR) and the webserver http://analysistools.nci.nih.gov/LDlink/ ^44^. Currently, a single variant is considered benign if the MAF is >5%^45^. Our findings suggest standards and guidelines could be revised to consider the combined impact of variants, particularly for more complex disorders such as albinism. Furthermore, the diagnosis of albinism currently focusses on compound mutations in single genes without considering the potential for synergistic relationships between functionally related genes such as that previously suggested for OCA2 and OCA3 genes (*OCA2* and *TYRP1*)^14^ and for which there is potentially one example in our cohort.

If our proposed tri-allelic genotype hypothesis is correct, this would increase the diagnostic yield of genetic testing from 22% as described earlier, to 56% in our cohort. Given that hypomorphic albinism is a difficult cohort to diagnose clinically, evidenced by the PAX6 mutation found in the atypical case (proband 9), further exome-seq is suitable for the genetic diagnosis. A sequencing technique with broad capture allows for the pickup of genetic variants which may have resulted in an overlapping ocular phenotype.

There is no current treatment for the underlying molecular anomaly in albinism and present treatments are supportive. Therapeutics are under development but an effective treatment for any of the underlying molecular defects has not yet reached clinical practice. Our work and that of others appears to suggest that small variations in melanin biosynthesis between related family members dictate the extent of the phenotype in OCA pedigrees. Furthermore, the net loss of TYR function (caused by cumulative effects of multiple variants, each of which reduce TYR function by differing amounts), appear to result in a continuum of clinical features.

Our work supports the assertion that small modulations in components of the melanin biosynthesis pathways, through therapeutic means, may be sufficient to rescue some of the visual disability seen in patients with albinism phenotypes.

## Electronic supplementary material

Supplementary Tables 1-3

## References

[1] Grønskov K, Ek J, Brondum-Nielsen K (2007). Oculocutaneous albinism. Orphanet J. Rare Dis..

[2] Thomas MG (2011). Structural grading of foveal hypoplasia using spectral-domain optical coherence tomography: a predictor of visual acuity?. Ophthalmology.

[3] Dorey S, Neveu M, Burton L, Sloper J, Holder G (2003). The clinical features of albinism and their correlation with visual evoked potentials. Br. J. Ophthalmol.

[4] McCafferty BK (2015). Clinical Insights Into Foveal Morphology in Albinism. J. Pediatr. Ophthalmol. Strabismus.

[5] Wolf, A. B., Rubin, S. E. & Kodsi, S. R. Comparison of Clinical Findings in Pediatric Patients With Albinism and Different Amplitudes of Nystagmus. *Journal of American Association for Pediatric Ophthalmology and Strabismus* **9**, 363–368, doi:http://dx.doi.org/10.1016/j.jaapos.2005.03.003 (2005). 10.1016/j.jaapos.2005.03.003 16102488

[6] Montoliu L (2014). Increasing the complexity: new genes and new types of albinism. Pigment Cell & Melanoma Research.

[7] Oetting, W. S. & King, R. A. Molecular basis of albinism: mutations and polymorphisms of pigmentation genes associated with albinism. *Hum. Mutat*. **13**, 99–115, doi:10.1002/(sici)1098-1004(1999)13:2<99::aid-humu2>3.0.co;2-c (1999). 10.1002/(SICI)1098-1004(1999)13:2<99::AID-HUMU2>3.0.CO;2-C 10094567

[8] King R (2003). Tyrosinase gene mutations in oculocutaneous albinism 1 (OCA1): definition of the phenotype. Hum. Genet..

[9] Hingorani M, Williamson KA, Moore AT, van Heyningen V (2009). Detailed ophthalmologic evaluation of 43 individuals with PAX6 mutations. Invest. Ophthalmol. Vis. Sci..

[10] Hutton SM, Spritz RA (2008). Comprehensive analysis of oculocutaneous albinism among non-Hispanic caucasians shows that OCA1 is the most prevalent OCA type. J. Invest. Dermatol..

[11] Gargiulo A (2011). Molecular and clinical characterization of albinism in a large cohort of Italian patients. Invest. Ophthalmol. Vis. Sci..

[12] Simeonov DR (2013). DNA Variations in Oculocutaneous Albinism: An Updated Mutation List and Current Outstanding Issues in Molecular Diagnostics. Hum. Mutat..

[13] Chiang P-W, Spector E, Tsai AC-H (2008). Evidence suggesting the inheritance mode of the human P gene in skin complexion is not strictly recessive. American Journal of Medical Genetics Part A.

[14] Chiang P-W, Fulton AB, Spector E, Hisama FM (2008). Synergistic interaction of the OCA2 and OCA3 genes in a family. American Journal of Medical Genetics Part A.

[15] Jagirdar K (2014). Molecular analysis of common polymorphisms within the human Tyrosinase locus and genetic association with pigmentation traits. Pigment cell & melanoma research.

[16] Mondal M, Sengupta M, Ray K (2016). Functional assessment of tyrosinase variants identified in individuals with albinism is essential for unequivocal determination of genotype to phenotype correlation. Br. J. Dermatol..

[17] Chiang PW, Spector E, Tsai AC (2009). Oculocutaneous albinism spectrum. Am. J. Med. Genet. A.

[18] Preising MN, Forster H, Gonser M, Lorenz B (2011). Screening of TYR, OCA2, GPR143, and MC1R in patients with congenital nystagmus, macular hypoplasia, and fundus hypopigmentation indicating albinism. Mol. Vis..

[19] Fukai K (1995). Autosomal recessive ocular albinism associated with a functionally significant tyrosinase gene polymorphism. Nat. Genet..

[20] Hutton SM, Spritz RA (2008). A Comprehensive Genetic Study of Autosomal Recessive Ocular Albinism in Caucasian Patients. Invest. Ophthalmol. Vis. Sci..

[21] Auton A (2015). A global reference for human genetic variation. Nature.

[22] Tripathi RK, Giebel LB, Strunk KM, Spritz RA (1991). A polymorphism of the human tyrosinase gene is associated with temperature-sensitive enzymatic activity. Gene Expr.

[23] Toyofuku K (2001). Oculocutaneous albinism types 1 and 3 are ER retention diseases: mutation of tyrosinase or Tyrp1 can affect the processing of both mutant and wild-type proteins. FASEB J..

[24] Chaki M (2011). Molecular and functional studies of tyrosinase variants among Indian oculocutaneous albinism type 1 patients. J. Invest. Dermatol..

[25] Oetting WS (2009). The R402Q tyrosinase variant does not cause autosomal recessive ocular albinism. American Journal of Medical Genetics Part A.

[26] Eichers ER, Lewis RA, Katsanis N, Lupski JR (2004). Triallelic inheritance: a bridge between Mendelian and multifactorial traits. Ann. Med..

[27] Li H (2009). The Sequence Alignment/Map format and SAMtools. Bioinformatics.

[28] Wang K, Li M, Hakonarson H (2010). ANNOVAR: functional annotation of genetic variants from high-throughput sequencing data. Nucleic Acids Res.

[29] Stenson PD (2014). The Human Gene Mutation Database: building a comprehensive mutation repository for clinical and molecular genetics, diagnostic testing and personalized genomic medicine. Hum. Genet..

[30] Ng PC, Henikoff S (2001). Predicting Deleterious Amino Acid Substitutions. Genome Res..

[31] Adzhubei IA (2010). A method and server for predicting damaging missense mutations. Nature methods.

[32] Cooper GM (2010). Single-nucleotide evolutionary constraint scores highlight disease-causing mutations. Nature methods.

[33] Chaki M, Mukhopadhyay A, Ray K (2005). Determination of variants in the 3’-region of the tyrosinase gene requires locus specific amplification. Hum. Mutat..

[34] Schouten JP (2002). Relative quantification of 40 nucleic acid sequences by multiplex ligation-dependent probe amplification. Nucleic Acids Res.

[35] Grønskov K (2009). Birth Prevalence and Mutation Spectrum in Danish Patients with Autosomal Recessive Albinism. Invest. Ophthalmol. Vis. Sci..

[36] Mondal M, Sengupta M, Samanta S, Sil A, Ray K (2012). Molecular basis of albinism in India: evaluation of seven potential candidate genes and some new findings. Gene.

[37] Opitz S, Käsmann‐Kellner B, Kaufmann M, Schwinger E, Zühlke C (2004). Detection of 53 novel DNA variations within the tyrosinase gene and accumulation of mutations in 17 patients with albinism. Hum. Mutat..

[38] Oetting WS, Fryer JP, Shriram S, King RA (2003). Oculocutaneous albinism type 1: the last 100 years. Pigment Cell Res.

[39] Dolinska MB (2016). Oculocutaneous Albinism Type 1: Link between Mutations, Tyrosinase Conformational Stability, and Enzymatic Activity. Pigment cell & melanoma research.

[40] Rooryck C (2008). Molecular diagnosis of oculocutaneous albinism: new mutations in the OCA1-4 genes and practical aspects. Pigment cell & melanoma research.

[41] Oetting WS (1991). Three different frameshift mutations of the tyrosinase gene in type IA oculocutaneous albinism. Am. J. Hum. Genet..

[42] Oetting WS, Garrett SS, Brott M, King RA (2005). P gene mutations associated with oculocutaneous albinism type II (OCA2). Hum. Mutat..

[43] Fryer JP, Oetting WS, King RA (2003). Identification and characterization of a DNase hypersensitive region of the human tyrosinase gene. Pigment Cell Res.

[44] Machiela MJ, Chanock SJ (2015). LDlink: a web-based application for exploring population-specific haplotype structure and linking correlated alleles of possible functional variants. Bioinformatics.

[45] Richards S (2015). Standards and guidelines for the interpretation of sequence variants: a joint consensus recommendation of the American College of Medical Genetics and Genomics and the Association for Molecular Pathology. Genet. Med..

[46] Kausar T, Bhatti M, Ali M, Shaikh R, Ahmed Z (2013). OCA5, a novel locus for non‐syndromic oculocutaneous albinism, maps to chromosome 4q24. Clin. Genet..

