# Supplementary material for: Identification of a functionally significant tri-allelic genotype in the Tyrosinase gene (TYR) causing hypomorphic oculocutaneous albinism (OCA1B)
Source: Sci Rep. 2017 Jun 30;7:4415. doi: 10.1038/s41598-017-04401-5 (PMC5493628; doi:10.1038/s41598-017-04401-5)
Supplement: Supplementary file 1 — Supplementary Tables 1-3 [file 41598_2017_4401_MOESM1_ESM.pdf]

Manuscript Title: **Identification of a functionally significant tri-allelic genotype in the Tyrosinase gene (*TYR*) causing hypomorphic oculocutaneous albinism (OCA1B).**

[1]Chelsea S Norman, [2]Luke O'Gorman, [3]Jane Gibson, [4]Reuben J Pengelly, [2]Diana Baralle, [1]J Arjuna Ratnayaka, [1]Helen Griffiths, [5]Matthew Rose-Zerilli, [6]Megan Ranger, [7, 2]David Bunyan, [1, 6]Helena Lee, [1]Rhiannon Page, [1]Tutte Newall, [6]Fatima Shawkat, [2, 8]Christopher Mattocks, [7]Daniel Ward, [4]Sarah Ennis, [1, 6]Jay E Self *

[1]Clinical and Experimental Sciences, Faculty of Medicine, University of Southampton

[2]Human Development and Health, Faculty of Medicine, University of Southampton

[3]Biological Sciences, Faculty of Natural and Environmental Sciences, University of Southampton

[4]Human Genetics & Genomic Medicine, Faculty of Medicine, University of Southampton

[5]Cancer Sciences Unit, Faculty of Medicine, University of Southampton

[6]Eye Unit, University Hospital Southampton

[7]Molecular Genetics Wessex Regional Genetics Laboratory, Salisbury NHS Foundation Trust

[8]Wessex Investigational Science Hub, University Hospital Southampton

Supplementary

	Median Coverage Across 18 Probands				Coverage at 20x																	
GENE	10x	20x	50x	100x	1	2	3	4	5	6	7	8	9	10	11	12	13	14	15	16	17	18
TYR	0.9971	0.9835	0.9333	0.7994	0.9788	0.9965	0.9864	0.9947	0.9971	0.9912	0.9794	0.9971	0.9971	0.9788	0.9971	0.9941	0.9953	0.9652	0.9805	0.9569	0.9593	0.9817
OCA2	0.9923	0.9923	0.9513	0.6317	0.9923	0.9923	0.9917	0.9923	0.9923	0.9923	0.9923	0.9923	0.9923	0.9923	0.9923	0.9923	0.9923	0.9907	0.9820	0.9867	0.9923	0.9923
TYRP1	0.9960	0.9750	0.8705	0.6053	0.9449	0.9824	0.9398	0.9886	0.9744	0.9852	0.9676	0.9654	0.9773	0.9648	0.9943	0.9807	0.9761	0.9455	0.9665	0.9211	0.9864	0.9546
SLC45A2	0.9949	0.9652	0.8536	0.5105	0.9339	0.9664	0.9550	0.9687	0.9960	0.9556	0.9595	0.9846	0.9635	0.9726	0.9954	0.9681	0.9664	0.9356	0.9595	0.9402	0.9385	0.9493
SLC24A5	0.9947	0.9947	0.9787	0.8017	0.9947	0.9947	0.9947	0.9947	0.9947	0.9947	0.9947	0.9947	0.9947	0.9947	0.9947	0.9947	0.9947	0.9947	0.9947	0.9947	0.9947	0.9947
C10orf11	0.8569	0.8569	0.8569	0.4000	0.8569	0.8569	0.8569	0.8569	0.8569	0.8569	0.8569	0.8569	0.8569	0.8569	0.8569	0.8569	0.8569	0.8569	0.8569	0.8569	0.8569	0.8569
GPR143	0.9024	0.8632	0.7806	0.3775	0.8754	0.8006	0.8746	0.8846	0.8597	0.8725	0.8647	0.8946	0.8561	0.7949	0.8796	0.8739	0.8739	0.8419	0.8953	0.8604	0.8006	0.8462
CACNA1F	0.9928	0.9903	0.9500	0.5993	0.9873	0.9867	0.9875	0.9917	0.9917	0.9863	0.9908	0.9928	0.9897	0.9845	0.9908	0.9910	0.9911	0.9897	0.9917	0.9891	0.9778	0.9815
PAX6	0.9929	0.9890	0.9261	0.7147	0.9760	0.9890	0.9877	0.9877	0.9883	0.9916	0.9870	0.9890	0.9903	0.9916	0.9922	0.9883	0.9903	0.9818	0.9916	0.9780	0.9741	0.9831

Supplementary table 1. Table indicating the median coverage for the 18 probands for 5 genes of interest. The coverage at 20X for the 5 genes of interest is shown for each of the 18 probands (numbered 1-18). Coverage is calculated based on the TruSight One target region.

Sample ID	Mean depth	Target bases 20x (%)
1	127.89	96.51
2	158.68	97.23
3	140.24	96.91
4	180.11	97.25
5	211.42	97.78
6	172.29	97.42
7	159.93	97.06
8	171.43	97.27
9	196.42	97.73
10	158.19	97.29
11	214.59	97.76
12	187.39	97.54
13	201.47	97.57
14	133.60	96.74
15	160.18	96.96
16	123.59	96.34
17	152.40	97.19
18	147.90	97.23

Supplementary table 2. Table indicating the mean depth and the 20X coverage for each sample. Coverage is calculated based on the TruSight One target region.

Gene Location	Forward Primer 5'- -3'	Reverse Primer 5'- -3'	Annealing Temperature	Product size (bp)
TYR exon 1*[15]	CATCTTCGATTTGAGTGCCC	CCCTGCCTGAAGAAGTGATT	59	521
TYR exon 4[33]	CTGTTTCCAATTTAGTTTTATAC	TACAAAATGGCCTATGTTAAGC	60	790

*Supplementary table 3. Primers used in PCR amplification. *Only a fragment of the exon due to the large size of exon 1.*
